# Grain Microstructure in Friction-Stir-Welded Dissimilar Al/Mg Joints of Thin Sheets with/without Ultrasonic Vibration

**DOI:** 10.3390/ma17194874

**Published:** 2024-10-04

**Authors:** Jialin Yin, Jie Liu, Chuansong Wu

**Affiliations:** MOE Key Laboratory for Liquid-Solid Structure Evolution and Materials Processing, Institute of Materials Joining, Shandong University, Jinan 250061, China; yinjialin@mail.sdu.edu.cn (J.Y.); 202134172@mail.sdu.edu.cn (J.L.)

**Keywords:** friction-stir welding, Al alloy, Mg alloy, ultrasonic vibration, grain microstructure, dynamic recrystallization

## Abstract

Electron backscattered diffraction (EBSD) characterization was conducted on the typical regions in friction-stir-welded dissimilar Al/Mg joints of 2 mm thick sheets with/without ultrasonic assistance. The effects of ultrasonic vibration (UV) on the grain size, recrystallization mechanisms, and degree of recrystallization on both sides of the Al-Mg bonding interface and the intermetallic compounds (IMCs) were investigated. It was found that on the Mg side of the weld nugget zone (WNZ), the primary dynamic recrystallization (DRX) mechanisms were discontinuous dynamic recrystallization (DDRX) and continuous dynamic recrystallization (CDRX), with geometric dynamic recrystallization (GDRX) playing a secondary role. On the Al side of the WNZ, CDRX was identified as the primary mechanism, with GDRX as a secondary contributor. While UV did not significantly alter the DRX mechanisms in either alloy within the WNZ, it promoted the aggregation and rearrangement of dislocations. This led to an increase in high-angle grain boundaries (HAGBs) and an enhanced degree of recrystallization in the welds. The average grain size in both the Al and Mg alloys of the WNZ followed a pattern of initially increasing and then decreasing along the thickness direction, reaching a maximum in the upper-middle part and a minimum at the bottom. The influence of UV on the average grain size in the WNZ was minimal, with only slight grain refinement observed, and the minimum refinement degree was only 0.9%. The Schmid factor (SF) on the WNZ and thermo-mechanically affected zone (TMAZ) boundary regions of the advancing side (AS) indicates that the application of UV increased the likelihood of basal slip and extension twinning in the crystal structure. In addition, UV reduced the thickness of IMCs and improved the strength of the Al-Mg bonding interface. These results suggest a higher probability of fracture along the TMAZ and WNZ boundary on the AS when UV was applied.

## 1. Introduction

In the context of global efforts towards energy conservation, emission reduction, and the development of a green economy, reducing structural weight has become a key development trend in the manufacturing industry. Al alloys and Mg alloys, as the two most commonly used lightweight metallic materials, are increasingly essential materials for critical structural components in industries such as high-speed rail, new energy vehicles, and electronic communication devices [1,2]. Simultaneously, using hybrid components of Al-Mg thin sheets in certain structures to complement the advantages of both alloys and further exploit their lightweight potential has become a significant focus in current manufacturing processes. For example, in the design of new energy vehicle battery case, in order to further realize the lightweight requirements, some companies have used Mg alloys to replace some Al alloys parts, and there is a demand for high-quality Al-Mg joints.

However, due to the inherent differences in the crystalline arrangements and physical–chemical properties of Al and Mg alloys, achieving high-quality Al-Mg joints remains a considerable challenge [3].

FSW offers advantages such as low heat generation, minimal weld defects, and high mechanical performance, making it an increasingly important method for joining similar or dissimilar Al/Mg alloys [4]. During the FSW process, the interaction between the shoulder and the tapered threaded pin, combined with the simultaneous rotation and advancement of the welding tool, results in a highly non-uniform thermal-mechanical coupling process characterized by intense plastic deformation [5]. This extreme and uneven thermal-mechanical coupling induces complex thermal processing across various regions, leading to varying degrees of dynamic recovery (DRV) and DRX. Consequently, distinctive microstructural features are formed in the weld, which significantly impact the mechanical performance of the welded joint. Based on the microstructural characteristics of different weld regions, the weld can be categorized into four distinct zones: the weld nugget zone (WNZ), the thermo-mechanically affected zone (TMAZ), the heat-affected zone (HAZ), and the base metal (BM) [6].

Prangnell et al. [7] adopted the technology of “stop action + freeze” in the friction-stir welding of 1 in thick Al-2195 plates with a spindle and traverse speed of 180 rpm and 1.7 mm/s to observe the microstructure evolution of the material when it met the rotating tool, and found that the grain refinement in the WNZ was caused by grain subdivision and the geometric effect strain, and the higher-temperature latter stages of the refinement process were similar to GDRX seen in high-strain hot torsion experiments. Liu et al. [8] carried out an FSW experiment of β-type Ti-15-3 alloy plate with a thickness of 1.45 mm at a rotation speed of 250 rpm and a welding speed of 50 mm/min, and found that the dislocation mobility in the TMAZ region was poor due to low stacking fault energy, and the microstructural evolution was mainly driven by DDRX. However, in the WNZ, CDRX mainly occurred because the high temperature and high strain enhanced the mobility of dislocations. S. Mironv et al. [9] studied the microstructure evolution of friction-stir-welded joints of 4 mm thick sheets of AZ31 Mg alloy in a wide range of rotation speeds from 300 to 3000 rpm, and maintained a welding speed of 200 mm/min, and found that the evolution of the grain structure was significantly affected by the very strong {0001}<uvtw> B-texture, which led to the general reduction in grain boundary misorientation during deformation.

For Al-Mg dissimilar joints, the formation of brittle-hard phase Al-Mg intermetallic compounds (IMCs) is a key factor affecting the properties of the junction [10]. Additionally, the evolution and distribution of the grain structure in the joint are crucial determinants of its overall performance [11]. In order to further control the microstructure evolution of the joint, enhance the strength of the joint, and improve the welding quality and efficiency, various modified FSW technologies have been proposed by researchers, including adding intermediate layers, such as Zn and Ni [12,13,14]; water cooling and liquid nitrogen cooling [15,16]; laser assistance [17,18]; electrical assistance [19,20]; and ultrasonic assistance [21,22,23,24]. All of these methods can be effective if the FSW process parameters, such as rotation speed and feed rate, are already optimized, as they significantly impact the metallurgical and mechanical properties of the joint. For instance, Farhang et al. [25] investigated the effects of these parameters on the residual stress, microstructure, and mechanical properties of the weld zone. In another study, Zhang et al. [26] characterized the nugget performance as influenced by rotational speed. Furthermore, UV, as a form of mechanical energy, can reduce the flow stress of metal, promote the plastic flow of materials, and inhibit the development of IMCs without changing the heat input, which makes UV a viable method for assisting FSW in Al-Mg dissimilar welds to achieve high-quality joints [27,28,29].

However, the introduction of an ultrasonic-assisted energy field will further influence the evolution of grain microstructure in different regions, thereby affecting the mechanical properties of the joint. Therefore, clarifying the mechanism of the ultrasonic-assisted energy field at the grain scale is of great theoretical and practical significance, which can contribute to a deeper understanding of microstructure evolution, regulate weld formation, and ultimately improve the service performance of the joints. Baradarani et al. [30] investigated the influence of ultrasonic assistance on the microstructure and texture evolution of AZ91 Mg alloy in FSW, and found that UV increased the proportion of HAGBs, and promoted the dynamic recovery and CDRX process, thus enhancing the degree of grain refinement. Hu et al. [31] carried out ultrasonic-assisted FSW experiments on 2219-T6 Al alloy with a 5 mm thickness. Through EBSD test of the welds, it was found that the effect of ultrasonic assistance on recrystallized grain evolution depended on the welding thermal cycle. Ultrasonic assistance promoted the grain boundary sliding and migration and the expansion frequency of local HAGBs, which significantly promoted the nucleation rate and growth rate of equiaxed grains during DRX. Zhao et al. [32] studied the DRX behavior of 3 mm Al-Mg dissimilar FSW/UVeFSW joints, and found that the ultrasonic energy field changed the main mode of DRX on the Mg side, which changed from CDRX to DDRX. Ultrasonic assistance enhanced the DRX process by promoting dislocation entanglement, aggregation, and rearrangement. The previous research shows that the introduction of UV promotes dislocation movement, promotes the dynamic recovery and recrystallization, refines grains, and even changes the main mode of DRX. However, the research focuses on similar Al alloys or Mg alloys, while research on the microstructure evolution of thin-plate dissimilar Al-Mg alloy joints is still lacking. This paper, based on the research on the evolution of the microstructure in various regions of 2 mm dissimilar joints, attempts to establish the microscopic mechanism of UV changing the fracture region of joints.

For 2 mm thick plates, compared with the commonly studied welding process of medium-thick plate with a thickness of 3 mm or more, a tool with relatively smaller shoulder size and shorter pin length was employed in the welding process, resulting in reduced heat generation and a weaker driving effect on the interface material. Correspondingly, the volume of the WNZ was smaller. With the variation in the thermal-mechanical coupling effect in the welding process, the microstructure evolution of the grains will also be significantly different from that in the medium-thick sheet joints. When UV is applied, the influence of UV on the grain microstructure in the weld will also change due to the change in the thermal-mechanical coupling effect between the tool and the workpiece. Therefore, it is of great guiding importance to characterize the grain microstructure of each area of Al/Mg joints with 2 mm thickness in FSW/UVeFSW and clarify the mechanism of UV in the microstructure evolution, which will promote the further application of the Al-Mg thin plate composite structure in the manufacturing industry.

In this work, 2 mm Al-Mg dissimilar alloy sheets were butt welded by FSW/UVeFSW, and the grain microstructure in different areas of weld cross-sections under typical process parameters was explored. The grain size, angular grain boundary, DRX mechanism and recrystallization degree of different regions and IMCs in the weld of Al-Mg joints in the FSW/UVeFSW were analyzed and compared. The effect of UV on the grain microstructure, IMCs, and fracture position of Al-Mg joints with 2 mm thickness was studied and the mechanism of the ultrasonic-assisted energy field on grains was clarified.

## 2. Experimental

**Figure 1:**
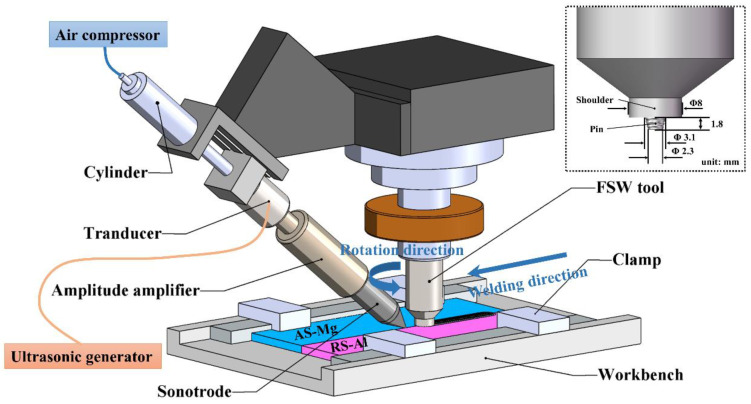
Schematic diagram of ultrasonic vibration-enhanced friction-stir welding (UVeFSW).

Conventional friction-stir welding (FSW) and ultrasonic vibration-enhanced friction-stir welding (UVeFSW) of 6061-T6 Al alloy and AZ31B-H24 Mg alloy sheets with 2 mm thickness were carried out. The experimental set-up is shown in Figure 1. The dimensions of Al/Mg sheets were 200 × 70 × 2 (length × width × thickness, unit: mm). The longitudinal direction was the rolling direction of the plate, that is, the welding direction. As shown in Figure 1, the cylinder pressure provided by the air compressor pushed the sonotrode to smoothly contact the surface of the base material. The angle between the sonotrode and the horizontal plane was 40°, and the distance from its contact point on the base material to the pin’s center was 20 mm. The ultrasonic generator operated at a frequency of 20 kHz, with a maximum amplitude of approximately 50 μm. The amplitude and ultrasonic power could be adjusted via the amplitude output ratio module on the control panel, within a range of 50% to 100%. For this experiment, the amplitude output ratio was set to 50%, resulting in a working amplitude of around 25 μm and an ultrasonic power of 260 W ± 10 W.

The tool, crafted from H13 tool steel, featured a concave shoulder with an 8 mm diameter. The right-handed thread pin had a length of 1.8 mm, with a root diameter of 3.1 mm and tip diameter of 2.3 mm. Figure 1 shows the shape and size of the tool. During welding, the tool rotated counterclockwise, with the Mg alloy sheet positioned on the advancing side. The tool was offset by 0.4 mm to the Mg alloy side, with a plunge depth of 0.15 mm and a tilt angle of 2°. The rotation speed of the tool was 1200 rpm, and its traverse speed was 50 mm/min.

After welding, the representative samples were cut by a wire cutting machine, followed by sandpaper grinding and mechanical polishing. A Leica Ion Beam Milling System EM TIC 3X (Leica Microsystems, Wetzlar, Germany) was used to mill the sample surface. A Zeiss Gemini 500 Field Emission Scanning Electron Microscope (Carl Zeiss AG, Oberkochen, Germany) and an Oxford Symmetry EBSD System probe (Oxford Instruments plc, Abingdon, UK) were used to collect electron backscattered diffraction signals. Scanning electron images were collected by a JEOL JSM-7800 Scanning Electron Microscope (JEOL(BEIJING) Co., Ltd., Beijing, China) and energy dispersive spectroscopy (EDS) line scanning analysis was undertaken by an Oxford Instruments XMax80-EDS (Oxford Instruments plc, Abingdon, UK).

**Figure 2:**
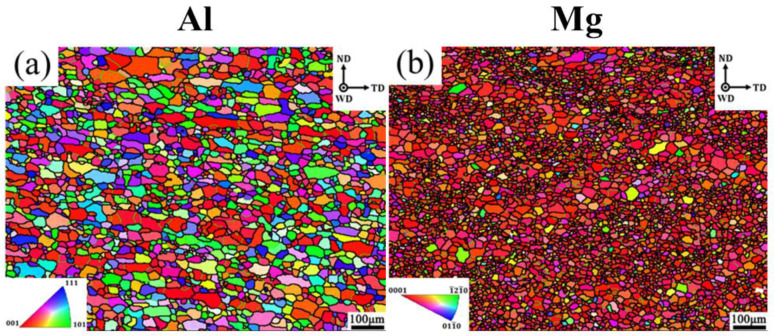
IPF maps of base metals—(**a**) Al alloy; (**b**) Mg alloy.

Figure 2a,b show the inverse pole figure (IPF) of the 6061-T6 and AZ31B-H24 base materials. For 6061-T6 alloy, the grain presents a weak “strip” orientation, and the grain length direction was the rolling direction of the plate. Within the scope of the observed area, the maximum grain size is 103.8 μm, the minimum grain size is 1.69 μm, and the average grain size is 20.32 μm. For AZ31B-H24 alloy, the maximum grain size is 61.10 μm, the minimum grain size is 1.70 μm, and the average grain size is 9.46 μm, which is a typical hot-rolled Mg alloy grain microstructure.

## 3. Results and Discussion

**Figure 3:**
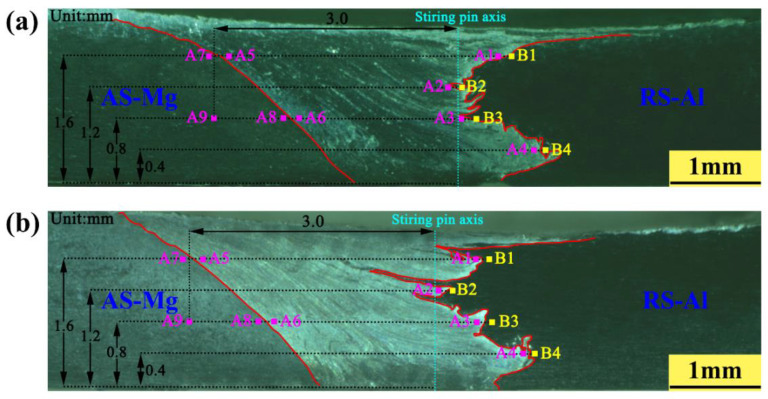
EBSD observation positions of the FSW/UVeFSW joint cross-section: (**a**) FSW; (**b**) UVeFSW.

The observation positions of EBSD characterization for FSW and UVeFSW joints are illustrated in Figure 3a,b, respectively. A1–A4 and B1–B4 are located on the Mg side and Al side of the bonding interface, A5–A8 are at the junction of the WNZ and TMAZ, and A9 is situated in the HAZ on the advancing side. Although the morphology of the Al-Mg bonding interface is not exactly the same in FSW and UVeFSW, the same depth at the transverse cross-section is selected for corresponding locations, which are as close as possible to the Al-Mg boundary. Additionally, for convenience in the following discussion, “C” denotes the conventional FSW process, while “U” refers to the UVeFSW process.

### 3.1. Grain Microstructure in WNZ

FSW is a typical thermo-mechanical coupling process. The friction between the tool and the workpiece generates heat, accompanied by the severe plastic deformation of the materials in the weld [33]. The grains in the weld will undergo dynamic recovery (DRV) and dynamic recrystallization (DRX), which will significantly affect the microstructure evolution and mechanical properties of the joint. In the FSW process of Al-Mg dissimilar alloys, several factors contribute to the complexity of the process due to the following reasons: (1) the distinct physical–chemical properties of the two materials to be welded; (2) the differing stress states on the advancing side (AS) and the retreating side (RS); and (3) the varied thermal-mechanical effects of the shoulder, the tapered thread pin, and the materials at different depths. These factors resulted in extremely non-uniform thermal-mechanical effects across different positions in the weld during FSW process.

The application of UV further complicates this process, as it differentially impacts the microstructure evolution in various regions, thereby changing the mode and extent of DRX. To investigate the influence of UV on the grain microstructure evolution at varying weld depths on both sides of the bonding interface, four positions on the Mg side (A1–A4) and four positions on the Al side (B1–B4) were selected along the weld depth direction of the Al-Mg dissimilar FSW/UVeFSW joint cross-section, as illustrated in Figure 3.

#### 3.1.1. A1–A4 Regions in the WNZ of the Mg Side

**Figure 4:**
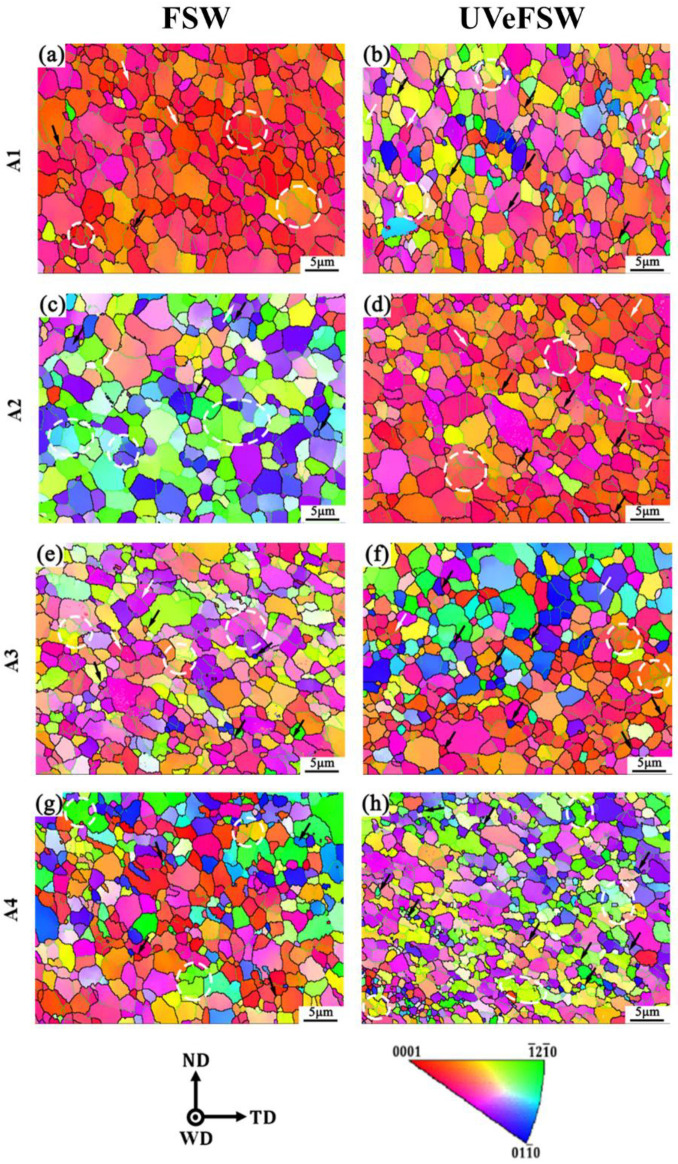
IPF maps of grain microstructures at the locations A1–A4 (Mg side) in FSW/UVeFSW welds (1200 rpm—50 mm/min). (**a**,**c**,**e**,**g**): FSW; (**b**,**d**,**f**,**h**): UVeFSW; (**a**,**b**): A1; (**c**,**d**): A2; (**e**,**f**): A3; (**g**,**h**): A4. (Black arrows: DDRX; White arrows: GDRX; White circle: CDRX.)

Figure 4 illustrates the IPF maps of A1-A4 regions in the WNZ of the Mg side. Except for Figure 4c,h, the grain orientation of A1–A4 regions is predominantly parallel to the <0001> direction, because there is a strong basal texture inside the Mg alloy. The grains are generally equiaxed, with no obvious deformation orientation observed. In addition, the IPF maps suggest that the UV has no discernible effect on the grain shape.

Figure 4 reveals the presence of numerous fine grains, which is undoubtedly formed by DRX during FSW process. The occurrence of DDRX in the Mg alloy is indicated by the black arrows in the images. The DDRX mechanism involves a distinct nucleation-growth process, where nucleation preferentially occurs through the bugling of grain boundaries at the triple junction between the original grain boundaries, followed by a long-distance migration of HAGBs [34]. In addition, GDRX is observed in A1, A2, and A3 regions, as shown by the white arrows in the images. The key feature of GDRX is the decomposition of elongated large grains into several subgrains, with low-angle grain boundaries (LAGBs) forming between these subgrains and minimal grain misorientation [35]. Concurrently, CDRX of some larger grains is observed in A1–A4 regions. CDRX is characterized by large regions of subgrains, typically featuring more LAGBs between them and often surrounded by grains with HAGBs outside the subgrains, as indicated by the white dotted circles in the images [36]. CDRX generally occurs in high stacking faulty energy (SFE) metals which are prone to cross-slip [37]. Although Mg alloy is generally considered as a low SFE metal, the SFE of the non-basal plane of Mg alloy is 4–7 times higher than that of the basal plane. Therefore, when the cross-slip of dislocations in the grains is activated, nucleation can also occur via the CDRX mechanism. To sum up, the grains of Mg alloy at the Al/Mg interface in the WNZ were affected by three DRX mechanisms: DDRX, CDRX, and GDRX, with DDRX and CDRX being dominant. The application of UV did not change the DRX mode of Mg alloy during the FSW process.

For FSW welds, the average grain sizes of A1–A4 regions are 2.36 μm, 2.43 μm, 1.95 μm, and 1.80 μm, respectively. For UVeFSW welds, the average grain sizes of A1–A4 regions are 2.11 μm, 2.32 μm, 1.92 μm, and 1.37 μm, respectively. The grain size increases from the A1 to A2 regions and then decreases, reaching a maximum in the A2 region and a minimum in the A4 region. By comparison, the application of UV resulted in an overall decrease in grain size, most notably in the A4 region, where the grain size was reduced by 23.89%. However, UV has minimal impact on grain size in other regions.

**Figure 5:**
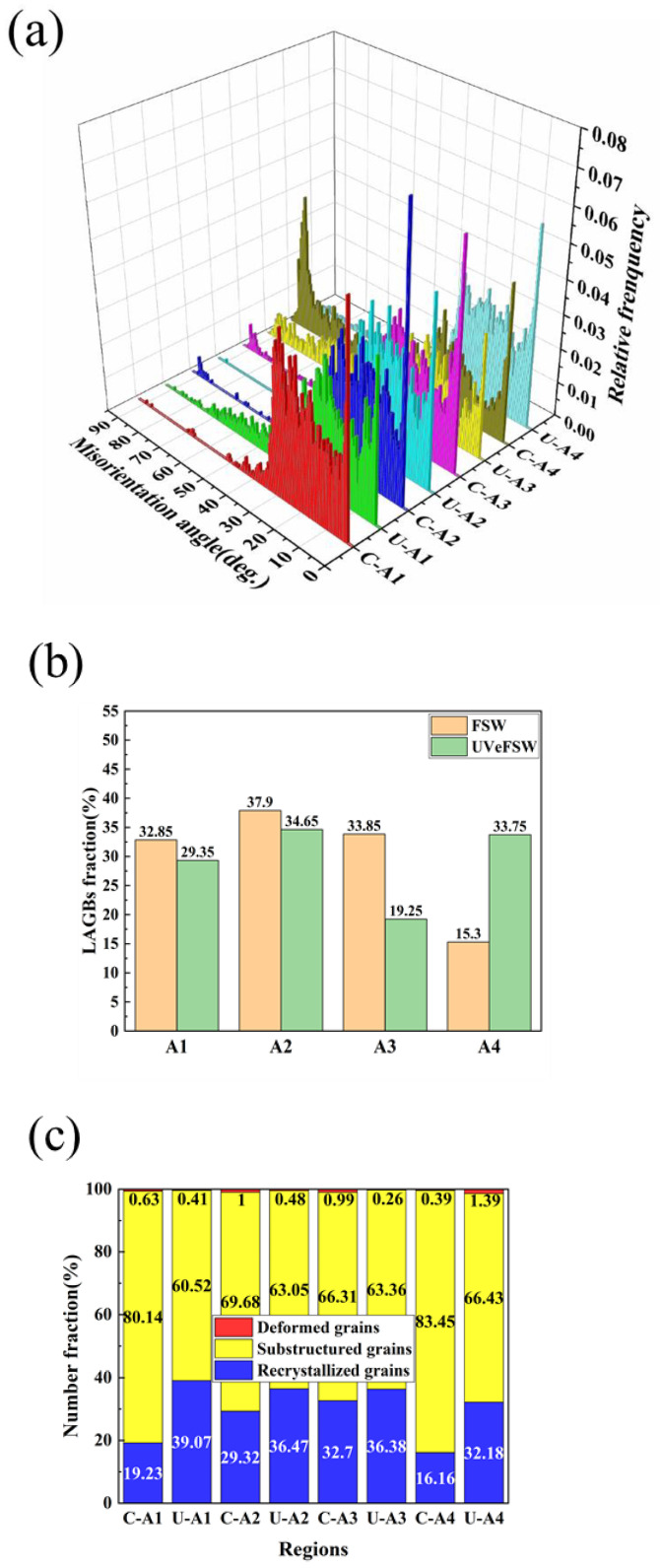
Grain microstructure information at locations A1–A4 (Mg side) in FSW/UVeFSW: (**a**) misorientation angle distribution, (**b**) LAGB fraction, (**c**) comparison of recrystallization degree.

Figure 5 shows the grain microstructure information of A1–A4 regions of welds in FSW/UVeFSW. Figure 5a indicates that in most A1–A4 regions, except U-A3 and C-A4 regions, the grain misorientation angles of Mg alloy in the WNZ primarily range between 2°and 40°, with a low proportion of grain misorientation angles exceeding 40°. In contrast, in the U-A3 and C-A4 regions, the grain misorientation angles are evenly distributed in the range of 0–90°. As shown in Figure 5b, the fraction of LAGBs in these two regions is relatively low, suggesting that DDRX is dominant in these two regions, while CDRX is dominant in other regions.

The data in Figure 5b show that from A1 to A4 regions, the proportion of LAGBs first increases and then decreases, and reaches the peak at A2 region. As can be seen from Figure 5c, the degree of recrystallization in the A2 region is also high. This indicates that severe CDRX occurred, resulting in a high fraction of LAGBs. These LAGBs absorbed dislocations and were then transformed into HAGBs, leading to the formation of recrystallized grains through the CDRX process. When UV was applied, the fraction of LAGBs in A1–A3 regions decreased and the degree of recrystallization increased, which shows that UV can effectively promote the DRX of Mg alloy grains. This enhancement may result from promoting CDRX or DDRX processes. In the CDRX mechanism, LAGBs gradually transform into HAGBs and form recrystallized grains. In the DDRX mechanism, a high fraction of LAGBs is not necessary. Instead, small grains with HAGBs form directly at grain boundaries or triple junctions of large grains. This also results in a decrease in the LAGB fraction and an increase in the recrystallization degree. As the IPF maps show in Figure 4, both of these methods may exist. For the A4 region, it has been mentioned that the fraction of LAGBs in the C-A4 region is low, the grain misorientation angles are evenly distributed within 0–90°, and the DDRX mechanism is dominant. Although the degree of recrystallization in this region is low, the grain size is the smallest, which is due to the lower temperature at the bottom of the WNZ and the slower grain growth rate. When UV was applied, the fraction of LAGBs increased, but the degree of recrystallization improved and the grain size was refined. This suggests that UV not only promoted the transformation of subgrains into recrystallized grains, but also facilitated the formation of more LAGBs in the recovery grains, which made preparations for subsequent recrystallization.

#### 3.1.2. B1–B4 Regions in the WNZ of the Al Side

**Figure 6:**
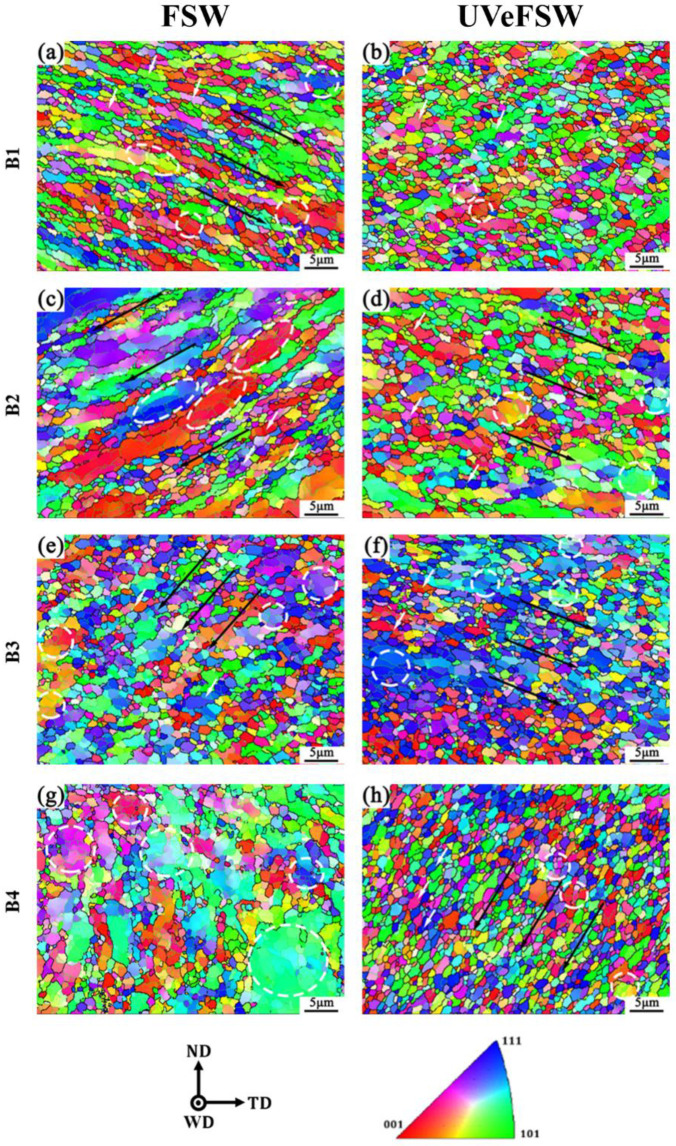
IPF maps of grain microstructures at the locations B1–B4 (Al side) in FSW/UVeFSW welds (1200 rpm—50 mm/min). (**a**,**c**,**e**,**g**): FSW; (**b**,**d**,**f**,**h**): UVeFSW; (**a**,**b**): B1; (**c**,**d**): B2; (**e**,**f**): B3; (**g**,**h**): B4. (Black arrows: DDRX; White arrows: GDRX; White circle: CDRX.)

Figure 6 illustrates the IPF maps of B1–B4 regions in the WNZ of the Al side. The B1 region is located 0.4 mm away from the workpiece surface, and the Al alloy grains in the B1 region in FSW show a significantly elongated strip-like shape with a downward flow deformation orientation, as shown by the long black arrow in Figure 6. This is because the Al alloy here was closer to the workpiece surface and was driven by the shoulder. However, elongated lath-like grains are not observed in the Al alloy in the B1 region in UVeFSW. The grains display a more regular equiaxed shape, suggesting that the application of UV influenced the material flow and DRX in this region.

The Al alloy in B2 and B3 regions was primarily influenced by the shearing deformation caused by the pin and the driving effect of the thread. Due to the right-handed thread of the pin and the tool counterclockwise rotation, the deformation orientation of the Al alloy grains in B2 and B3 regions inclines downward. However, the application of UV changed the original characteristics of flowing along the lower left to the lower right. In the B4 region, due to the small diameter of the tip of the pin, the material in this area was weakly driven by the pin, leading to equiaxed grains in the WNZ of the FSW weld. When UV was applied, the Al alloy grains in this area tended to flow towards the lower left. These indicates that UV enhanced the material flow in the WNZ.

The grains on the Al alloy side exhibit various degrees and types of DRX. In the B1–B3 regions of the WNZ in both FSW and UVeFSW, the Al alloy was subjected to shearing by the pin and driving by the thread, resulting in GDRX. This mechanism led to the decomposition of large grains with distinct deformation orientations into several subgrains with similar orientations, as indicated by the white arrow in Figure 6. When UV was applied, a combination of GDRX and CDRX characteristics was observed in the B4 region. The white dotted circle in Figure 6 highlights the region where CDRX was occurring, indicating that CDRX was the predominant DRX mechanism for Al alloy grains in this area. Different from Mg alloy grains, a large amount of DDRX occurred, and no obvious DDRX characteristics were observed in Al alloy grains. Although some grains nucleated at grain boundaries, most of them contained LAGBs, so they were not DDRX.

For the Al side of the FSW weld, the average grain sizes in B1–B4 regions are 1.07 μm, 1.11 μm, 1.03 μm, and 1.02 μm, respectively. For the Al side of the UVeFSW weld, the average grain sizes are 1.05 μm, 1.10 μm, 0.98 μm, and 0.99 μm, respectively. The grain size of the Al side is much smaller than that of the Mg side, but the grain size of the Al parent material is twice as large as that of the Mg parent material, which shows a more significant DRX phenomenon in Al alloy grains during FSW. Overall, when UV was applied, there is a general reduction in grain size across the B1–B4 regions. However, due to the already fine grain size of the Al alloy in the WNZ, the effect of UV on further grain refinement was relatively minor. The primary influence on the grain size of materials in the WNZ was the thermal-mechanical process of FSW, with UV only contributing to slight additional refinement.

**Figure 7:**
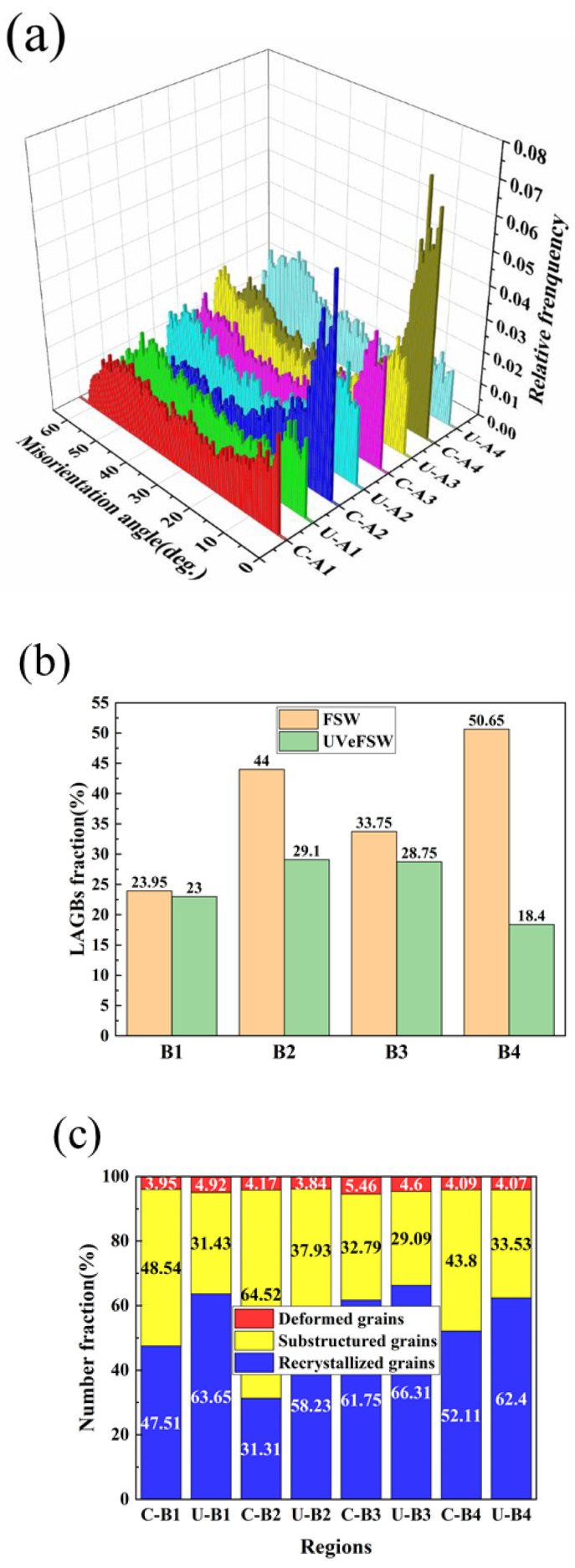
Grain microstructure information at locations B1–B4 (Al side) in FSW/UVeFSW: (**a**) misorientation angle distribution, (**b**) LAGB fraction, (**c**) comparison of recrystallization degree.

Figure 7 presents the grain microstructure information of B1–B4 regions of welds in FSW/UVeFSW. In these regions, the grain misorientation angles of Al alloy are mainly distributed within 2–60°. The misorientation angles initially decrease between 2° and 25° before increasing between 25° and 55°. Compared to the B1–B4 regions of the FSW weld, the fraction of LAGBs in the corresponding regions of the UVeFSW weld is reduced, indicating an increase in the degree of recrystallization. Notably, the proportion of LAGB in the U-B1 and U-B4 regions is very low, suggesting a high degree of DRX. Conversely, the proportion of LAGB in the C-B2 region is relatively high, corresponding to a lower degree of DRX. This observation further supports the conclusion that applying UV promoted CDRX in the Al alloy in the WNZ. The fraction of LAGBs exhibits a pattern of first increasing, then decreasing, and finally increasing among the B1–B4 regions of the FSW weld. The degree of recrystallization negatively corresponds to the trend of the LAGB, which also indicates that the occurrence of CDRX on the Al alloy side in FSW. It should be pointed out that the fraction of LAGBs of Al alloy in the WNZ is much lower than that of the Mg alloy counterpart, but the degree of recrystallization is much higher than that of the Mg alloy counterpart. Combined with the fact that the grain size of Al alloy is smaller than that of Mg alloy, this suggests the CDRX process in Al alloy is more thorough.

### 3.2. Grain Microstructure at the Junction between the WNZ and TMAZ of the Mg Side

The preliminary experiments show that the tensile specimens of the UVeFSW joint were fractured at the junction of the WNZ and TMAZ on the advancing side [38], so EBSD analysis at the A5–A8 regions of the junction between the WNZ and TMAZ was carried out.

**Figure 8:**
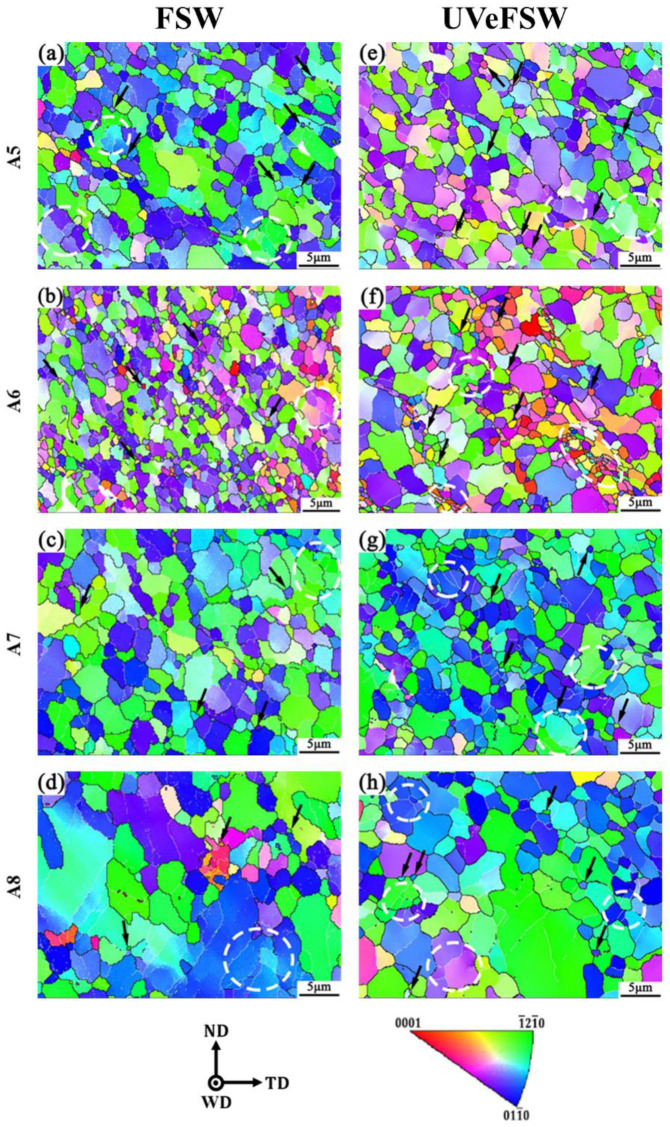
IPF maps of grain microstructures at the locations A5–A8 in FSW/UVeFSW welds (1200 rpm—50 mm/min). (**a**–**d**): FSW; (**e**–**h**): UVeFSW; (**a**,**e**): A5; (**b**,**f**): A6; (**c**,**g**): A7; (**d**,**h**): A8. (Black arrows: DDRX; White arrows: GDRX; White circle: CDRX.)

For the grains at A5 and A6 in Figure 8, obvious deformation orientation from upper left to lower right can be seen. This is mainly because the A5 region and A6 region were located at the side edge of the pin and the materials were strongly driven by the thread, showing the material flow from the top to the bottom in the WNZ. However, A7 and A8 regions were in the TMAZ, where the materials were not in direct contact with the pin, but were driven by the materials in the WNZ to flow to a certain extent. So, they show an obvious grain deformation orientation. It can be seen that the grains at A5–A8 regions are mostly parallel to <-12-10> or <01-10>. These grains are different from the grains on the Mg alloy side in the WNZ, which is basically parallel to <0001>; this indicates that the original basal texture has deviated. By observing IPF maps in Figure 8, it can be seen that DRX has also occurred in different ways in the A5–A8 regions, mainly CDRX represented by white circles and DDRX represented by black arrows in the images. No obvious GDRX is observed. This because the grains in the A5–A8 regions did not reach the critical point where GDRX occurred, even though some deformation occurred. As can be seen from the white dotted box in the lower right corner of Figure 8f, there is a large number of small grains with a lot of HAGBs and a small amount of LAGBs among them, which is a distinct feature of CDRX. It was speculated that UV promoted dislocation movement, which made a large number of dislocations concentrate on deformed grains. Then, the accumulated dislocations gradually transformed LAGBs into HAGBs, thus forming fine recrystallized grains.

For the FSW process, the average grain sizes of Mg alloy in the A5–A8 regions are 2.68 μm, 1.90 μm, 3.21 μm, and 4.06 μm, respectively. For the UVeFSW process, the average grain sizes of Mg alloy are 2.89 μm, 1.88 μm, 2.77 μm, and 4.07 μm, respectively. The grain sizes of Mg alloy in the A5–A8 regions are larger than those of Al alloy and Mg alloy on both sides of the Al/Mg interface in the WNZ, which is mainly due to the fact that the strain rate of materials in this region is lower than that in the WNZ, and the recrystallization degree of materials here is lower than that in the WNZ. The grain sizes of Mg alloys in the A5 and A6 regions are generally lower than those in the A7 and A8 regions. However, the percentage of recrystallization degree in these four regions is close. The reason for this is that A7 and A8 regions were located in the TMAZ, so some of the original larger grains in the base material were retained. The effect of UV on the grain size of Mg alloy in the A5–A8 regions is not significant.

**Figure 9:**
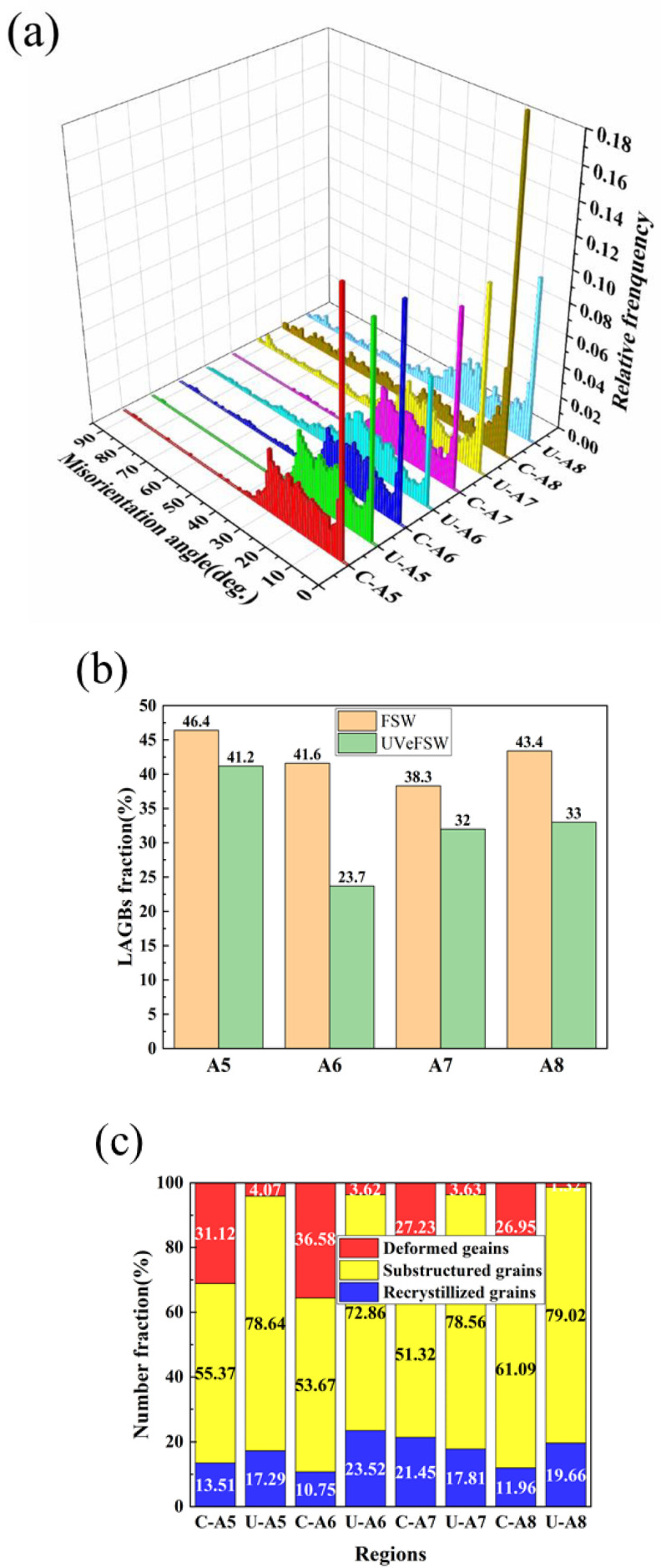
Grain microstructure information at locations A5–A8 in FSW/UVeFSW: (**a**) misorientation angle distribution, (**b**) LAGB fraction, (**c**) comparison of recrystallization degree.

Figure 9 shows the grain microstructure information in the A5–A8 regions in FSW/UVeFSW. In A5–A8 regions, except for C-A8, the grain misorientation angles are mainly distributed within 2–40°, and the peaks appear at 2° and 30°. However, the fraction of grain misorientation angles greater than 40° is very low, which is consistent with the distribution of misorientation angles on the Al alloy side in the WNZ. For C-A8, the grain misorientation only peaks at 2°, and is evenly distributed within 3–90°, but the fraction of LAGBs is high, so CDRX is still dominant. Combined with its large grain size, it shows that some original grains are still retained here, and the degree of recrystallization is low. The data in Figure 9b show that the UV also caused the fraction of LAGBs to decrease, the recrystallization degree to increase, and the average grain size to decrease slightly. This also shows that the application of UV can not only improve the recrystallization degree of the WNZ, but also improve the recrystallization degree of grains located on both sides of the interface between the TMAZ and WNZ on the AS. Figure 9c shows that when UV was applied, the fraction of deformed grains greatly decreased and the fraction of recovered grains greatly increased. For FSW, at the initial stage of welding, the original base metal undergoes severe plastic deformation under the strong drive of the shoulder and the pin, forming a large number of deformed grains, and the dislocation density increases greatly. In the subsequent process, due to a high strain rate and certain temperature, DRX occurs in the weld. Before the DRX is completed, dislocations will move to the inside of deformed grains or grain boundaries, which determined different DRX methods. Within the deformed grains, dislocations were continuously accumulated to form dislocation walls, that is, a multilateralization process. Then, large deformed grains split and formed subgrains. These subgrains were recovery grains, and the formation of recovery grains prepared for the subsequent transformation from LAGBs to HAGBs.

For A5–A8 regions, because the material here was only driven by the material in the WNZ region, the plastic deformation degree and the temperature were low, so the DRX degree was also low. The application of UV promoted the accumulation of a large number of dislocations into the deformed grains, which led to the multilateralization of the deformed grains and the formation of recovery grains. Furthermore, UV enhanced the transformation of recovery grains into recrystallization grains. As can be seen from Figure 9c, in the A5–A8 regions, UV promoted the transformation of deformed grains into recovery grains.

The plastic deformation of Mg alloy is mainly realized by slip and twinning. At room temperature, the plastic deformation ability of Mg alloys is poor because of less movable slip systems [39,40]. The critical resolved shear stress (CRSS) of the basal slip system of Mg alloy at room temperature is only 0.6–0.7 MPa, while the non-basal slip system is much larger than the basal slip system, so it is not easy to start at room temperature [40,41]. Another important plastic deformation mechanism of Mg alloy is twinning, which can coordinate the rotation of grains when Mg alloy is deformed and can play a role at different temperatures [42]. The CRSS required for Mg alloy twinning is about 2–3 MPa, and it is easy to start when plastic deformation occurs at room temperature.

The premise of crystal sliding is that the shear stress component acting on the sliding surface along the sliding direction is greater than the CRSS. In order to evaluate the possibility of slip and twinning of Mg alloy base material and an Al/Mg dissimilar friction-stir-welded joint with or without UV during tensile testing at room temperature, the Schmidt factor (SF) is introduced and compared. When the SF is large, it means that the grains are in soft orientation and there is a greater possibility of sliding. When the SF is small, it means that the grains are in hard orientation and the possibility of sliding is small.

**Figure 10:**
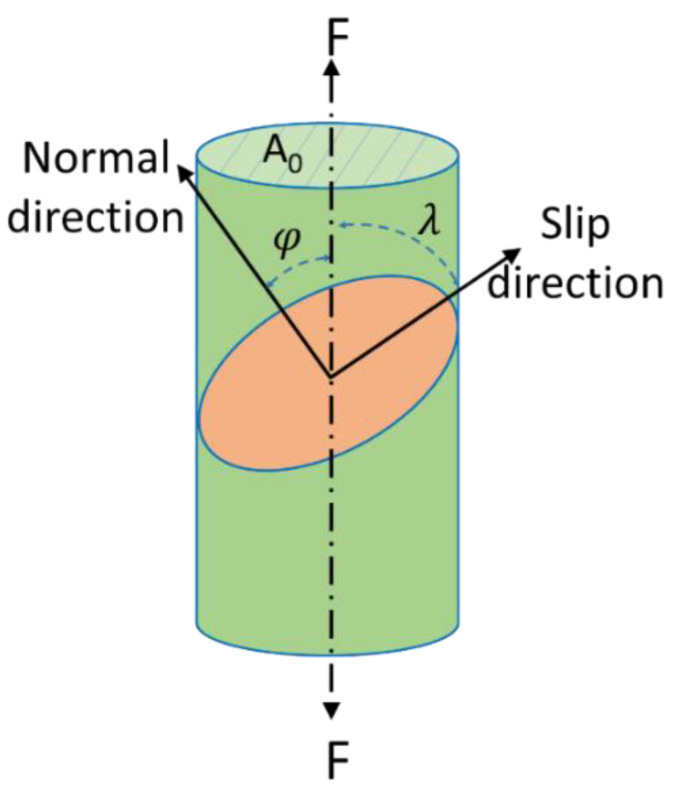
Schematic diagram of single crystal slip under uniaxial tension.

Figure 10 shows the slip diagram of the crystal grain under uniaxial tension. The angle between the slip direction and the applied force is λ, and φ is the angle between the normal to the slip plane and the applied force. $A_{0}$ is the normal cross-sectional area of the single crystal test bar, and F is the force applied to the crystal. In order to make the shear stress component of the applied force F acting on the slip surface along the slip direction greater than CRSS, the following relationship should be satisfied:

(1) $$\tau \geq \frac{Fcos\lambda }{\frac{A_{0}}{cos\varphi }}=\frac{F}{A_{0}}cos\lambda cos\varphi =\sigma cos\lambda cos\varphi$$

where cosλcosφ in Formula (1) is the numerical value of the SF.

**Figure 11:**
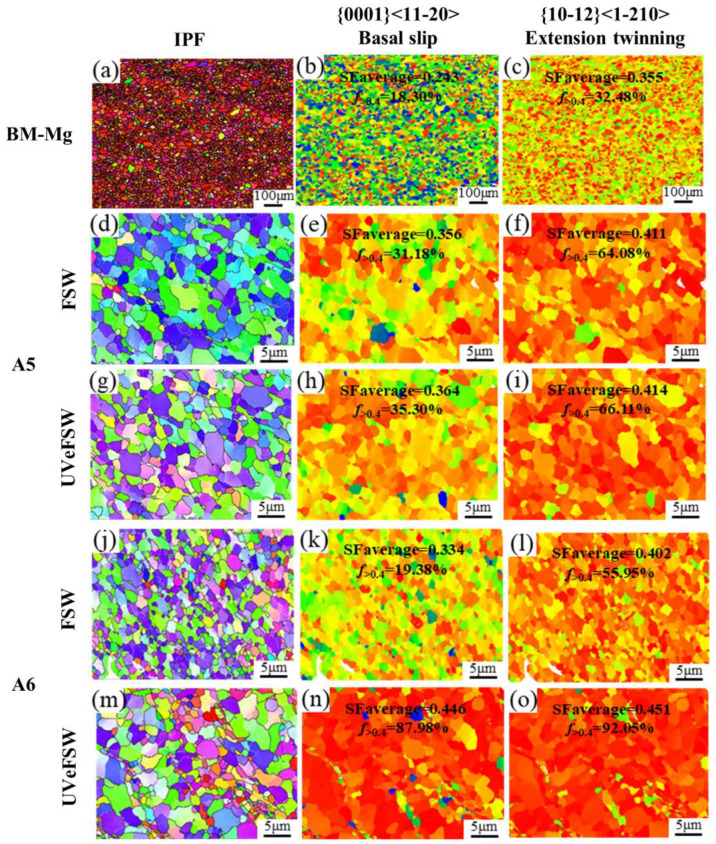

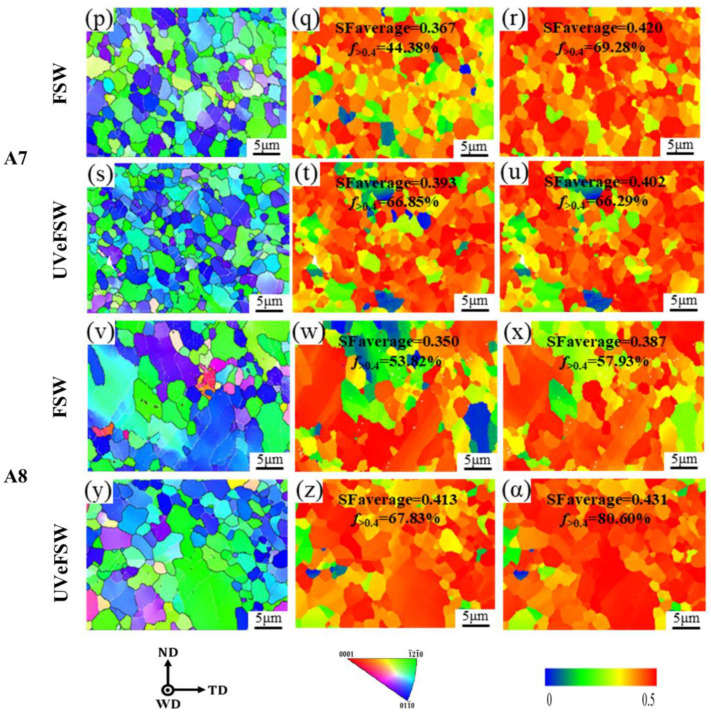
IPF maps and distribution maps of the Schmidt factor for Mg alloy base metal and locations A5–A8. BM-Mg: (**a**–**c**); A5:(**d**–**i**); A6: (**j**–**o**); A7: (**p**–**u**); A8: (**v**–**α**); FSW: (**d**–**f**,**j**–**l**,**p**–**r**,**v**–**x**); UVeFSW: (**g**–**i**,**m**–**o**,**s**–**u**,**y**–**α**).

The average SF of the basal slip system and extension twinning of the Mg parent metal and the boundary between the WNZ and TMAZ of AS was calculated by using Channel 5 (5.0.9.0) software, and the percentage of the SF values greater than 0.4 was determined. As can be seen from Figure 11, compared with the FSW joint, the base metal of Mg alloy has the lowest SF, which shows that the external stress required for the slip and twinning of the base metal during stretching is large, and the base metal has a larger yield strength. It can be found that the average SF of twinning in a tensile state is greater than that of the base slip system, which means that Mg alloy parent material is more prone to twinning in uniaxial tension. It can be seen from Figure 2b that the base material of Mg alloy has a strong basal texture, and the grain C-axis is parallel to the plate length direction. In uniaxial tension, the direction of the external force was parallel to the width direction of the plate, which led to the fact that when the original parent material was in tension, the basal plane was in hard orientation and did not easily slip; additionally, plastic deformation mainly depended on twinning, so the SF of extension twinning is much larger than that of the basal plane slip. When the Mg alloy base material underwent FSW, the average SF of the basal slip system and extension twinning of the weld increased relative to the base material, which means that the basal slip and extension twinning of the welded joint were more likely to occur, especially extension twinning, which also led to the obvious reduction in the yield strength of the welded joint relative to the base material.

For FSW/UVeFSW joints, the twinning mechanism played an extremely important role when plastic deformation occurred. Figure 11 shows that the average SF of A7 and A8 regions is larger than that of A5 and A6, which shows that the Mg alloy grains near the TMAZ were more likely to slip and twin. However, during the experiment, the actual fracture position of the UVeFSW joint was at the boundary between the TMAZ and the WNZ and near the nugget zone. The reason for this phenomenon is because there are more continuous IMC strips near the WNZ, which provides a path for the continuous crack propagation. By comparing the SF of the four areas A5–A8, it is found that the application of UV has a greater tendency to cause basal plane slip and extension twinning of crystals in this area, that is, when UV was applied, the welded joint was more likely to break along the boundary between the TMAZ and WNZ.

### 3.3. Grain Microstructure in the TMAZ of the Mg Side

**Figure 12:**
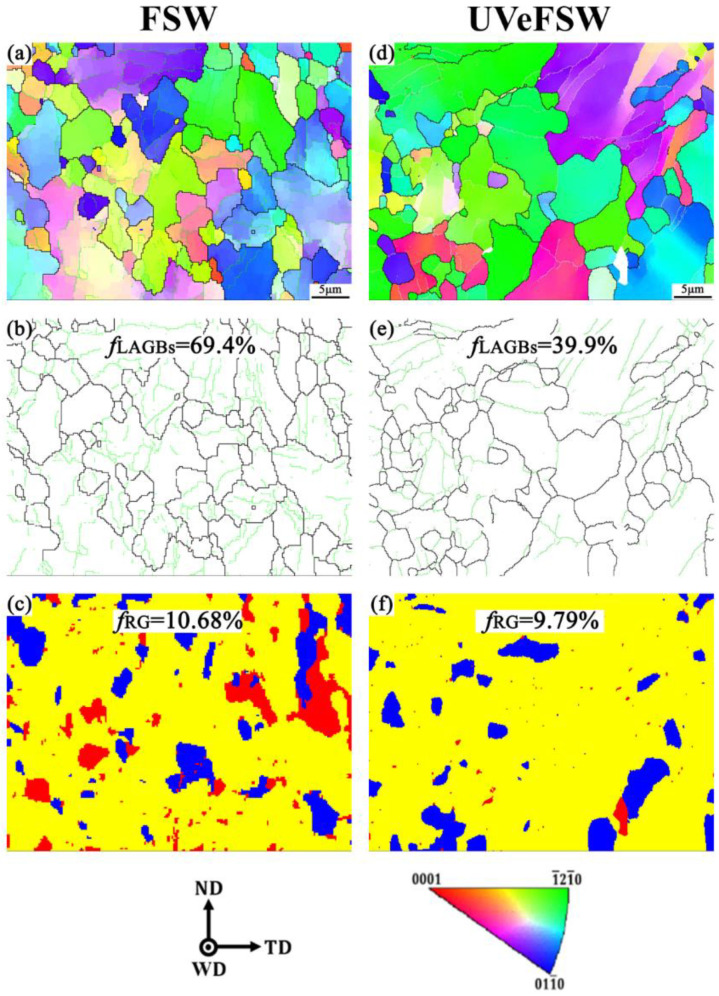
Grain microstructure information at the location TMAZ-A9 on AS (*n* = 1200 rpm, *v* = 50 mm/min)—(**a**,**d**): IPF maps; (**b**,**e**): GB maps; (**c**,**f**) recrystallization grain distribution maps; (**a**–**c**): FSW; (**d**–**f**): UVeFSW.

In order to observe the change in grain microstructure from the WNZ to the HAZ, the Mg alloy located on the AS was characterized by EBSD, and the scanning position was the A9 regions in Figure 3. Figure 12 shows the grain microstructure information of Mg alloy in the HAZ. It can be clearly seen from Figure 12a,d that the Mg alloy in the HAZ contains both larger grains and some smaller grains, but the grain size is much larger than that in the WNZ. The average grain size of the HAZ of the Mg side in FSW/UVeFSW is about 4.75 μm and 5.70 μm, respectively. Compared with the base metal grain size of Mg alloy, which is 9.46 μm, the grains in the HAZ reduced. This is probably because the heat generation of Al/Mg dissimilar FSW with a thickness of 2 mm was low, which caused the slow grain growth.

As shown in Figure 12b,e, compared with the low fraction of LAGBs of Mg alloy base material, which is about 11.5%, a large number of LAGBs were produced in the HAZ after FSW/UVeFSW. This shows that the Mg alloy material located in the HAZ underwent plastic deformation to a certain extent, resulting in dislocations and accumulating in the deformed grains. By comparison, it can be found that when UV was applied, the grain size in the HAZ increases slightly. The data in Figure 12c,f indicate that the recrystallization of Mg alloy was not obvious, with only about 10% recrystallized grains. This limited recrystallization is likely due to the relatively low temperature and plastic deformation experienced in the HAZ. It is important to note that, since the HAZ was distant from the sonotrode, the ultrasonic effect was weak, and no obvious signs of grain refinement were observed.

### 3.4. IMCs at the Bonding Interface

**Figure 13:**
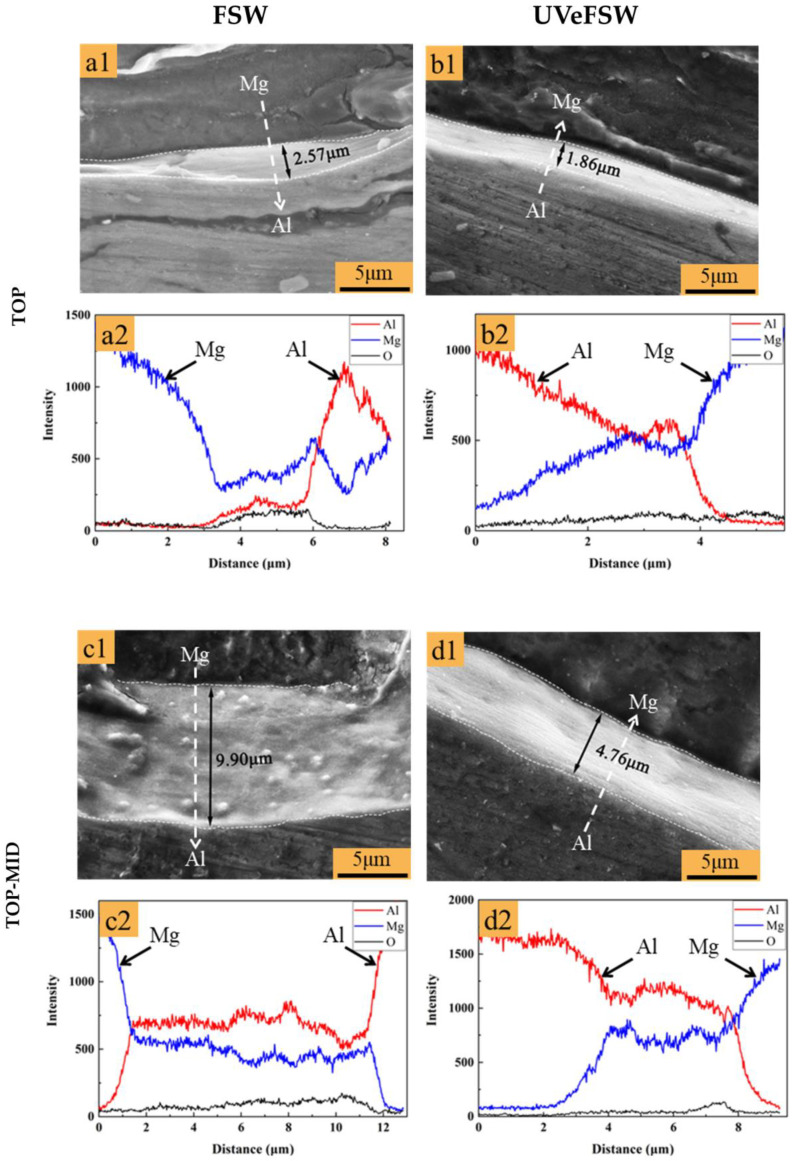

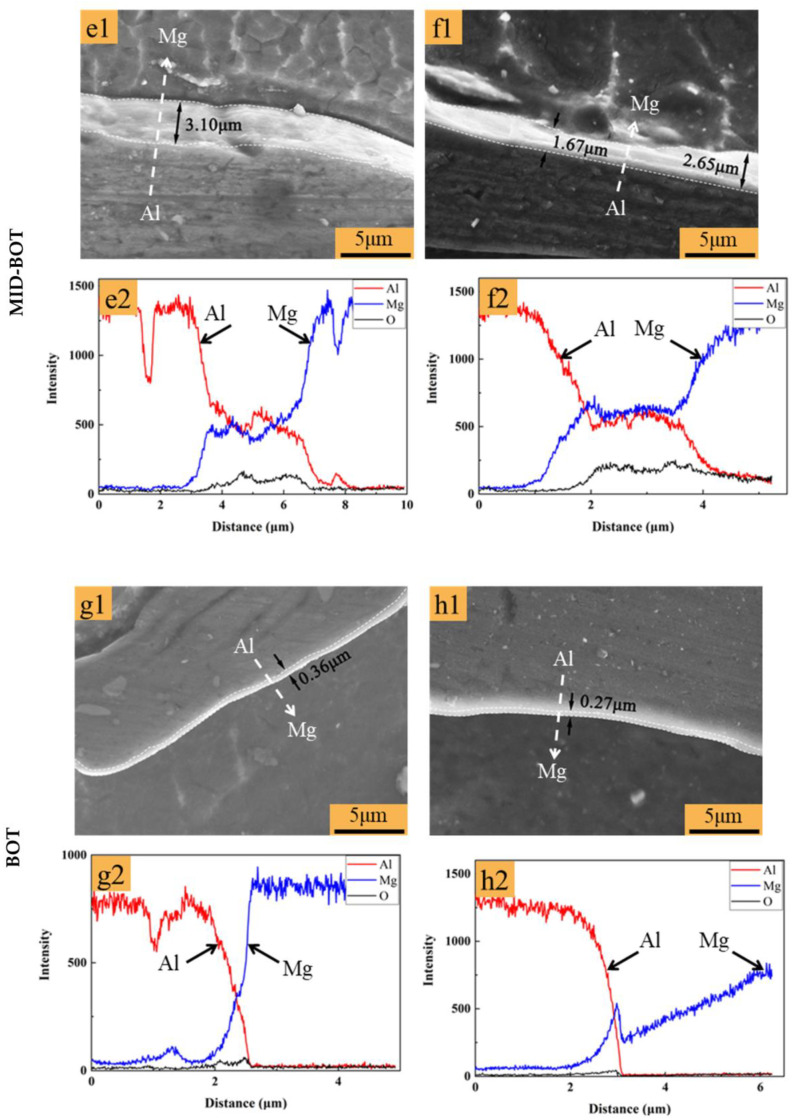
SEM images of the IMC layer and EDS results at four observation points on the cross-section of the FSW/UVeFSW weld (1200 rpm—50 mm/min). IMCs SEM image: (**a1**,**b1**,**c1**,**d1**,**e1**,**f1**,**g1**,**h1**); EDS line scanning: (**a2**,**b2**,**c2**,**d2**,**e2**,**f2**,**g2**,**h2**).

Four points between A1–A4 and B1–B4 at the bonding interface illustrated in Figure 3 were selected to conduct characterization of scanning electron microscopy (SEM) and energy dispersive spectroscopy (EDS). Figure 13 compares the SEM images of the IMC layer and EDS results at four observation points (TOP, TOP-MID, MID-Bot, and BOT) on the cross-section of the FSW/UVeFSW welds. For both FSW and UVeFSW, the IMC thickness at TOP-MID is largest. As the depth increases, the IMC thickness first rises and then decreases. This is because the different depths experience different thermo-mechanical actions, which determine the growth of IMCs. On the other hand, application of UV can decrease the IMC thickness at all four points. The average values of IMC thickness at four points are 3.9835 μm in FSW and 2.2625 μm in UVeFSW.

**Figure 14:**
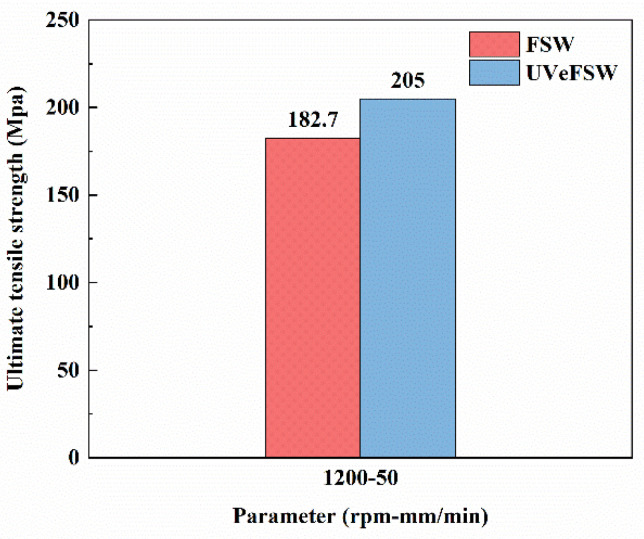
Ultimate tensile strength of the Al/Mg dissimilar joints FSW/UVeFSW (1200 rpm—50 mm/min).

Because exertion of UV enhances the mechanical interlocking (Figures 3, 6 and 7 in [38]), increases the recrystallization degree (Figure 9), and changes the fracture position from the bonding interface to the boundary between the WNZ and TMAZ (Figure 11 in [38]), the ultimate tensile strength (UTS) of the joints is increased from 182.7 MPa in FSW to 205 MPa in UVeFSW, as shown in Figure 14. This means that with application of UV, the strength of Al/Mg dissimilar joints is increased by 12.2%.

In previous research, Al-Mg dissimilar FSW improved the joint UTS more or less, but the extent of increase was less than that in this work. The UTS of AZ61 Mg alloy and 6061-T6 Al alloy joints reached 181.33 MPa by introducing a Zn interlayer [13]. In water cooling-assisted FSW, the UTS of an AA6061 and AZ31B joint with a thickness of 6 mm was 182 MPa [15]. For liquid nitrogen cooling-assisted FSW, Al-Mg joints with the highest UTS of 134 MPa were achieved for 3 mm thickness 5083 Al alloy and AZ31C-O Mg alloy [16]. Hybrid laser-FSW with a Ni foil interlayer was adopted to weld 6061-T6 and AZ31 with a thickness of 4 mm, and a dissimilar joint with a UTS of 169 MPa was obtained [17]. Additionally, a joint with a UTS of 120 MPa can be attained through ultrasonic-assisted stationary shoulder FSW of 3 mm thickness 6061-T6 Al alloy and AZ31B Mg alloy [23]. In this work, the UTS of the Al-Mg dissimilar joints with a thickness of 2 mm can reach up to 205 MPa with the application of UV. The reason for this is that the application of UV promoted the degree of DRX in various regions of the joint without obviously changing the heat input, and played a certain role in grain refinement. Furthermore, UV inhibited the growth of brittle IMCs, resulting in thinner IMCs at the bonding interface. This reduced the likelihood of stress concentration and enhanced the interfacial bonding strength. Additionally, UV improved the plastic deformation tendency in the area near the WNZ-TMAZ boundary, thus changing the fracture position of the Al-Mg dissimilar joint and enhancing the UTS.

## 4. Conclusions

(1) For the dissimilar FSW joints of Al/Mg sheets, the materials in the WNZ exhibit obvious dynamic recrystallization at certain temperatures and severe plastic deformation. For Mg alloy in the WNZ, the main DRX mechanisms were DDRX and CDRX, supplemented by GDRX. For Al alloy in the WNZ, the main DRX mechanism was CDRX, supplemented by GDRX.

(2) The application of UV does not obviously change the DRX mode of Al alloy and Mg alloy in the WNZ, but can reduce the fraction of LAGBs and increase the fraction of HAGBs by promoting CDRX or DDRX, which indicates that UV promotes the DRX in the WNZ to a greater extent.

(3) Although Al alloy and Mg alloy in the WNZ have undergone different grain microstructure evolution, their average grain sizes show a trend of increasing first and then decreasing along the thickness direction, reaching the maximum in the mid-upper part and the minimum at the bottom. While UV has a limited effect on the average grain size of the Al and Mg alloys in the WNZ, a slight grain refinement is observed.

(4) A comparison of the SF of A5–A8 reveals that the application of UV at the junction of the WNZ and TMAZ makes the crystal structure in this region more prone to slip and twinning. This suggests that when UV was applied, the welded joint is more likely to fracture along the boundary between the WNZ and the TMAZ. This conclusion is consistent with the previous experimental results in UVeFSW.

(5) Through SEM-EDS characterization of IMCs at the interface, it is found that the thickness of IMCs at the interface decreases with the application of UV, which improves the strength of the Al-Mg bonding interface, changes the fracture position of the joint, and improves the UTS of the Al-Mg dissimilar joint.

## Data Availability

The original contributions presented in the study are included in the article, further inquiries can be directed to the corresponding author.
